# AI awareness and employee boundary-spanning behavior: a nonlinear mechanism

**DOI:** 10.3389/fpsyg.2026.1952833

**Published:** 2026-10-07

**Authors:** Zihe Zhou, Ping Wang

**Affiliations:** Department of Human Resource Management, School of Economics and Management, Zhejiang Sci-Tech University, Hangzhou, China

**Keywords:** AI awareness, AI help-seeking behavior, AI job crafting, Boundary-spanning behavior, conservation of resources theory

## Abstract

Drawing on conservation of resources theory, this study develops a nonlinear model of the association between AI awareness (AIA) and employee boundary-spanning behavior (BSB). It examines the mediating role of AI job crafting (AIC) and the joint moderating roles of AI help-seeking behavior (AIHSB) and leader AI job crafting (LAIC). Using two-wave time-lagged matched survey data from 501 employees of accounting firms, this study finds that: (1) AIA has strict U-shaped relationships with both BSB and AIC; (2) AIC exhibits a level-dependent nonlinear mediating pattern in the relationship between AIA and BSB; (3) AIHSB significantly moderates the U-shaped relationship between AIA and AIC, while LAIC further moderates this moderating effect. The conditional indirect effect varies jointly with AIA and the two moderators. This study advances research on AIA from divergent linear effects to a level-dependent nonlinear transition. It identifies the boundary conditions jointly created by individual access to AI resources and leader role modeling. The findings inform how organizations can help employees adapt to AI-driven change.

## 1 Introduction

From ChatGPT, Claude, and Gemini to AI agents capable of managing email, scheduling, and workflow coordination, artificial intelligence is rapidly evolving from an information-generation tool into an infrastructure for task execution and process coordination. Its consequences now extend beyond efficiency gains to the deeper reorganization of jobs, skills, and organizational governance (AI Index Steering Committee, 2025; World Economic Forum, 2025). The “AI Plus” initiative has appeared in the State Council’s Government Work Report for three consecutive years (State Council of the People’s Republic of China, 2024, 2025, 2026), while the policy emphasis has shifted from continued promotion to broader, deeper deployment. Large-scale adoption in priority industries and the diffusion of new forms such as AI agents are accelerating. AI is therefore no longer merely a macro-level technological trend; it has become a concrete feature of organizational workflows, collaborative relationships, and employees’ career development.

As AI becomes embedded in organizations, it does more than alter how employees perform their tasks. It prompts them to reassess their occupational position, the value of their existing capabilities, and their future career prospects. AI awareness (AIA) captures employees’ subjective assessment of how intelligent technologies may affect their job security, career development, and future opportunities (Brougham and Haar, 2018; Liang et al., 2022). Such awareness can intensify job insecurity (Lingmont and Alexiou, 2020) and induce withdrawal through emotional exhaustion (Teng et al., 2024). Yet it may also stimulate work engagement (Ding, 2021), proactive learning (Gui et al., 2024), and job crafting (Kang et al., 2023), thereby eliciting more adaptive responses, including the search for resources across organizational boundaries (Faulconbridge et al., 2025). AI awareness is thus associated not only with employees’ attitudes and emotions but also with their decisions about task adjustment, resource acquisition, and collaboration. It may therefore be associated with their willingness to develop external connections and engage in boundary-spanning behavior (BSB).

Boundary-spanning behavior refers to employees’ efforts to establish and maintain ties with key actors outside their immediate work unit to obtain the information, feedback, support, and resources needed to accomplish unit goals (Ancona and Caldwell, 1992). In conventional organizational settings, such behavior is widely viewed as a mechanism for accessing external knowledge, integrating heterogeneous resources, and strengthening team adaptability (Edmondson and Harvey, 2018). Under AI-driven change, however, employees confront not only adjustments within their assigned tasks but also the reconfiguration of knowledge boundaries, collaborative partners, and resource channels. The effect of AI awareness on boundary-spanning behavior may therefore be nonlinear. As AI awareness rises from low to moderate levels, employees increasingly perceive risks of job displacement, skill depreciation, and uncertainty in collaboration. Because boundary spanning requires additional time, relationship maintenance, and coordination, employees may initially protect their remaining resources by concentrating on established tasks and familiar relationships. At higher levels of AI awareness, however, employees may recognize that preserving existing knowledge structures, task boundaries, and modes of collaboration creates even greater risks of maladaptation and resource loss. Boundary spanning then ceases to be merely an additional burden and becomes a means of securing external information, relational support, and heterogeneous resources. Employees may consequently move beyond established work boundaries to reconstruct their adaptation strategies through external ties. Different levels of AI awareness may thus activate distinct resource appraisals and behavioral choices, producing a nonlinear relationship.

Existing research has not adequately addressed this possibility. First, studies of AI awareness have concentrated largely on psychological and attitudinal outcomes, including job insecurity, turnover intentions, job satisfaction, and career competence (Kong et al., 2021; Lingmont and Alexiou, 2020). Although recent work has considered adaptive behaviors such as proactive learning and job crafting (Ding, 2021; Kang et al., 2023), it has paid less attention to whether employees move beyond established job and unit boundaries to mobilize external resources and relational networks. Examining boundary-spanning behavior therefore extends research toward a more outward-facing and action-oriented form of adaptation to AI. Second, although prior research has shown that AIA may be experienced as both threatening and challenging, this literature has largely framed the issue as a linear contrast between facilitation and inhibition. It has paid less attention to whether changes in AIA intensity produce a directional shift in proactive adaptation—that is, a nonlinear effect. This study therefore moves beyond whether AIA is beneficial or harmful and examines whether its behavioral associations depend on its level. BSB provides an appropriate setting in which to investigate this question because it is an externally oriented form of proactive adaptation that requires additional time and coordination.

The behavioral response to AI awareness is also unlikely to involve an immediate, simultaneous increase or decrease in boundary spanning. Employees may first assess how AI relates to their tasks, workflows, and capability requirements; infer its implications for job value, access to resources, and career development; and then decide whether to modify their task arrangements, collaborative practices, and work-related cognitions. This process constitutes AI job crafting (AIC). AI job crafting refers to employees’ proactive modification of AI-related tasks, interactions, and cognitions to achieve a more effective fit between the employee and AI (He, 2025; Li et al., 2024). It converts the perception of external technological pressure into internal adjustment of the job. Job crafting comprises cognitive, relational, and task crafting (Wrzesniewski and Dutton, 2001). In an AI context, cognitive crafting entails redefining the goals, boundaries, and sources of value associated with work after AI adoption. This reinterpretation can lead employees to recognize that information within their formal role is no longer sufficient and to seek resources and contacts beyond existing boundaries. Relational crafting involves actively reconfiguring collaborative networks around AI and broadening the sources and scope of relational resources. Employees may reach out to people who can help them understand, use, and adapt to AI, thereby directing attention toward actors outside the work unit who can provide support, information, and collaborative opportunities. Task crafting entails reallocating work content and effort in response to AI-enabled changes to tasks—for example, delegating standardized, repetitive tasks to AI while devoting more attention to core activities and cross-boundary collaboration (Law and Varanasi, 2025; Li et al., 2024). Once task resources are reorganized, employees may depend more heavily on knowledge, cooperation, and support beyond their formal roles. AI job crafting therefore constitutes a central mechanism through which AI awareness may be associated with boundary-spanning behavior. It reflects how subjective assessments of technological disruption may be associated with proactive adjustments to tasks, relationships, and cognition, thereby changing how employees acquire resources and build external connections. This mechanism helps explain both why AI awareness may have nonlinear effects and how those effects may be transmitted to boundary-spanning behavior.

Accordingly, this study uses conservation of resources (COR) theory as the overarching framework and examines AIA, AIC, and BSB within a single analytical model. We focus on the nonlinear effects of AIA on AIC and BSB, AIC’s role in transmitting these nonlinear associations, and the boundary conditions provided by individual resource acquisition and leader role modeling. AIA is an employee’s subjective assessment of technological change and its career consequences, but this assessment alone may not fully activate BSB. Its translation into behavior involves at least three linked questions. First, will employees redesign their own work—how will they change their work? This question concerns AIC. Compared with general job crafting, AIC directly addresses changes in tasks, knowledge, and human–AI collaboration that arise when AI enters the workplace; it therefore captures more precisely how employees convert a broad assessment of AI-related disruption into concrete work adjustments. Li et al. (2024) likewise define AIC as domain-specific job crafting in AI contexts and identify it as an important form of proactive adaptation to AI-induced changes in work. Second, can employees obtain the information, methods, and feedback needed to support such adjustments—what resources will they use to change their work? This question concerns AI help-seeking behavior (AIHSB), which reflects an employee’s proactive tendency to use AI to obtain informational, methodological, and feedback resources (Lee, 1997; Geller and Bamberger, 2012). Employees who are more willing to seek help from AI can draw on richer cognitive, informational, and problem-solving resources when confronting AI-related disruption, making them more likely to translate AIA into AIC. Third, are these adjustments feasible and behaviorally legitimate in the local work context—under what conditions will employees change their work? This question concerns leader AI job crafting (LAIC), which captures leaders’ role-modeling behavior as they proactively adjust tasks, collaboration, and cognition in response to AI. Such crafting not only signals organizational support (He et al., 2023), but also provides employees with a behavioral reference for understanding and using AI, thereby increasing the likelihood that they will engage in AIC with the support of AIHSB (Li et al., 2024). The proposed framework integrates three levels—behavioral translation, individual resources, and leader-provided context—to better explain how BSB develops in AI-enabled work settings.

## 2 Theoretical background and hypotheses

### 2.1 AI awareness and boundary-spanning behavior

Boundary-spanning behavior consists of activities directed beyond the employee’s immediate work unit. Its defining feature is the establishment of external contacts, exchange of information, and coordination of cooperation to obtain the feedback and support required for task accomplishment (Ancona and Caldwell, 1992). Because these activities require substantial time, relationship maintenance, and role coordination, they do not arise automatically in response to environmental change. Rather, they depend on whether employees are willing to bear the additional costs and believe that crossing boundaries will generate sufficient returns (Marrone et al., 2007). Employees’ willingness to move beyond existing boundaries therefore rests heavily on how they evaluate the associated gains and losses of resources.

At the onset of AI awareness, employees are likely to focus first on uncertainties surrounding job displacement, skill depreciation, and constrained career prospects (Brougham and Haar, 2018; Liang et al., 2022). Under these conditions, BSB becomes costly, requiring employees to invest additional time and attention in relationship maintenance and coordination. A resource-protection orientation may therefore suppress their willingness to develop external ties (Marrone et al., 2007). Employees are more likely to preserve existing skills, competence, familiar routines, stable relationship networks, and career security than to cross boundaries to seek new contacts and collaboration. As technological restructuring intensifies and AIA rises, however, employees may gradually recognize that information and resources within their existing roles are no longer sufficient to meet emerging work demands. Continuing to defend established boundaries may then imply greater future losses, including skill obsolescence, lost opportunities, and misaligned relationship networks (Faulconbridge et al., 2025). Boundary spanning then acquires a different meaning: rather than an additional burden, it becomes an avenue for acquiring heterogeneous resources and reducing future career risks (Ancona and Caldwell, 1992; Marrone et al., 2007). Employees who interpret AI-driven change as an opportunity to cross boundaries or coordinate across domains are more likely to engage in boundary-crossing innovation, interdisciplinary collaboration, and cross-boundary cooperation (Faulconbridge et al., 2025). By contrast, when AI is interpreted primarily as a signal of diminished agency and compressed boundaries, employees are more inclined to preserve existing boundaries (Yan and Chen, 2025). The effect of AI awareness on boundary-spanning behavior should therefore shift across levels: resource defense predominates in the early stage. As the perceived impact intensifies, employees gradually shift toward resource expansion by learning, experimenting, searching more extensively for external information, and investing in boundary-spanning relationships.

*H1*: AI awareness has a U-shaped relationship with employee boundary-spanning behavior. As AI awareness increases, boundary-spanning behavior first decreases and then increases.

### 2.2 AI awareness and AI job crafting

The introduction of AI into the workplace requires employees to reassess not only tools but also career trajectories, capability structures, and job boundaries. Early research conceptualized STARA awareness as employees’ perceptions that smart technologies may replace human labor (Brougham and Haar, 2018). Subsequent research has extended AIA beyond a singular perception of threat to a conceptual framework in which gains and depletion coexist (Gui et al., 2025). Research on challenge–hindrance appraisals likewise suggests that employees’ appraisals of AI may be associated with different outcomes through job crafting, job insecurity, and AI knowledge (He et al., 2024; Sha and Chai, 2025). Together, these studies indicate that the behavioral consequences of AIA are inherently complex.

Conservation of resources theory holds that individuals strive to retain, protect, and accumulate valued resources. When resources are actually or potentially threatened, individuals tend to adopt defensive strategies to prevent further loss (Hobfoll et al., 2018). Yet when sufficient resources are available—or when external change can no longer be avoided—they may invest resources to generate new gains and restore environmental fit (Hobfoll, 1989; Hobfoll et al., 2018). AI job crafting is a form of person–task–technology adjustment that requires employees to reconfigure task, relational, and cognitive boundaries (He, 2025; Li et al., 2024). This process consumes cognitive and emotional resources and exposes employees to the costs of experimentation and possible failure (Hobfoll, 1989; Hobfoll et al., 2018). Accordingly, as AI awareness rises from low to moderate levels, the principle of resource-loss primacy suggests that employees will initially interpret AI-driven change as an erosion of existing resources rather than as an opportunity to acquire new ones (Hobfoll et al., 2018). Perceived risks of displacement, skill depreciation, and declining career prospects become salient threats to resources (Brougham and Haar, 2018; Liang et al., 2022). In this state, individuals prioritize protecting their time, attention, existing skills, competence, and familiar work routines. They consequently reduce AIC, which requires learning, experimentation, and reconstructing tasks, relationships, and cognition (Hobfoll et al., 2018; Hobfoll, 1989). Because AIC entails tangible costs, it may be negatively associated with AIA during the initial stage of resource threat.

This inhibitory tendency should not persist indefinitely. As AIA rises to a high level, the expected resource loss associated with inaction may exceed the short-term costs of learning, experimentation, and work redesign. Employees may then invest in AI skills, learning time, and relational resources, and reconfigure their tasks, collaboration, and work-related cognition to maintain career competitiveness (Hobfoll et al., 2018; Hobfoll, 1989). When employees appraise AI-related change as a challenge or opportunity, promotion-oriented job crafting becomes more pronounced. Conversely, when employees primarily appraise AI as a hindrance or threat, the threat itself does not automatically become beneficial. Rather, the expected loss associated with maintaining the status quo increases and prevention-oriented and avoidance-based crafting is more likely (Ding, 2021; Xu et al., 2026). As AI awareness increases, employees’ job-crafting response should therefore shift from resource defense to resource investment rather than rise or fall monotonically. This transition implies an initial decline followed by an increase.

*H2*: AI awareness has a U-shaped relationship with employee AI job crafting. As AI awareness increases, AI job crafting first decreases and then increases.

### 2.3 The mediating role of AI job crafting

AI awareness may affect boundary-spanning behavior not only through employees’ interpretations of career uncertainty but also through whether those interpretations are translated into proactive changes to the job itself. Rather than adjusting their boundary-spanning behavior immediately and in parallel with AI awareness, employees may first reinterpret their work, revise relationships, and redesign tasks. AI job crafting constitutes this work-proximal process of behavioral translation. Prior studies show that AI awareness may be associated with employees’ reassessment of the fit between their work practices and the technological environment and, in turn, with proactive task, relational, and cognitive crafting (Gui et al., 2024; Kang et al., 2023). AI job crafting also extends beyond the formal job by encouraging employees to reconsider the resources, information, and collaborators required for task accomplishment, thereby creating conditions for subsequent movement across established boundaries (Li et al., 2024; Liu et al., 2026a). As employees continually redesign their work in response to AI, their boundary-spanning activity may intensify (Faulconbridge et al., 2025). Generative AI can enable employees to delegate peripheral tasks to technology and redirect attention toward complex problem solving and cross-boundary collaboration; task, relational, and cognitive crafting jointly facilitate this transition (Law and Varanasi, 2025). AI job crafting should therefore account for part of the association of AI awareness to boundary-spanning behavior. Organizational behavior research has begun to examine mediating mechanisms within curvilinear relationships. For example, Zhao et al. (2023) found that work engagement mediates the U-shaped relationship between perceived overqualification and innovative behavior. This finding suggests that when the relationship between a predictor and an outcome is nonlinear, the transmission process should be interpreted conditionally across different levels of the predictor. Drawing on H1 and H2, we therefore formulate H3 as a level-dependent nonlinear statistical mediation hypothesis:

*H3*: AIC mediates the nonlinear relationship between AIA and BSB. Specifically, the instantaneous indirect effect linking AIA to BSB through AIC varies across AIA levels.

### 2.4 The moderating role of AI help-seeking behavior

AIA reflects an employee’s subjective assessment of technological change and its career consequences, but the same degree of perceived AI impact does not necessarily elicit the same work adjustments. Whether employees can translate ambiguous technological uncertainty into identifiable and manageable task problems depends on the problem-solving resources available to them. Help-seeking research shows that when employees’ ability to solve problems independently is constrained, they can obtain information, methods, and cognitive support by seeking help, thereby closing the gap between existing knowledge and task requirements (Lee, 1997; Geller and Bamberger, 2012). As AI becomes embedded in work processes, it also becomes a repeatedly accessible source of problem-solving support that provides immediate feedback and involves fewer interpersonal costs than conventional help-seeking.

Our concern is therefore not whether AI-related disruption induces employees to seek help, but how employees’ existing use of AI resources alters the translation of AIA into AIC. When AIHSB is high, employees can continually obtain explanations, possible solutions, and feedback from AI. Abstract perceptions of disruption can then be decomposed into concrete work-adjustment problems, and repeated experimentation can help employees refine their work practices and gradually develop both the capacity and the willingness to redesign their work (Fröhlich et al., 2017). When AIHSB is low, employees may recognize that AI could change their work yet still lack actionable information; their AIA is therefore more likely to remain at the level of threat appraisal. In this study, AIHSB thus represents a boundary condition concerning resource availability rather than a mediating process assumed to follow inevitably from AIA. We accordingly propose:

*H4*: AIHSB positively moderates the U-shaped relationship between AIA and AIC. As AIHSB increases, the positive curvature of the relationship between AIA and AIC becomes stronger, and the U-shaped pattern becomes more pronounced.

### 2.5 The moderating role of leader AI job crafting

#### 2.5.1 Leader AI job crafting as a contextual condition for the moderating effect of AI help-seeking

Although AI help-seeking behavior may alter the relationship between AI awareness and AI job crafting, this moderating effect does not operate independently of context. Even when employees obtain information and feedback from AI, they must still assess whether the information should be adopted, whether proactive adjustment is feasible, and whether the organization will accept such adjustment. Social learning theory proposes that individuals evaluate the feasibility, appropriateness, and desirability of a behavior by observing significant others and the consequences of their actions (Bandura, 1977). Leaders are therefore important contextual referents for determining what employees are allowed to do, what is worth doing, and what the organization will accept. COR theory and social learning theory therefore play complementary roles in the present framework. COR theory explains why employees conserve or invest resources in response to AI-related disruption, whereas social learning theory explains how leader behavior provides contextual cues about whether such resource investment is feasible and legitimate. In an AI context, leader AI job crafting provides a salient modeling cue: it demonstrates that actively revising task arrangements, collaborative practices, and cognitive frames in response to AI is both feasible and encouraged. Consistent with this argument, leaders’ attention to and investment in AI enhance employees’ readiness for change (He et al., 2023).

Leader AI job crafting is not merely a background condition for AI help seeking; it may shape whether and how the resources obtained through AI help seeking are translated into employees’ AI job crafting. When leader AI job crafting is low, employees who frequently seek help from AI may still lack behavioral models, contextual support, and perceived legitimacy, making it difficult to translate AI-generated information and feedback into concrete job redesign. When leaders themselves actively craft their work around AI, however, employees can use leaders’ behavior as a reference, and resources acquired through AI help seeking are more readily activated and converted into action (Li et al., 2024). Employees are then more likely to interpret AI disruption as an external change that requires active adaptation rather than as a career threat to be endured.

We therefore expect LAIC and employee AIHSB to jointly condition the nonlinear relationship between AIA and AIC, although the joint moderation may differ across combinations of these variables. Building on H4, we propose:

*H5*: LAIC positively moderates the moderating effect of AIHSB on the U-shaped relationship between AIA and AIC. As LAIC increases, AIHSB more strongly amplifies the positive curvature of this relationship.

#### 2.5.2 The conditional indirect effect of AI help-seeking and leader AI job crafting

The mediating role of AI job crafting is unlikely to remain equally strong across contexts. It should vary with employees’ means of resource acquisition and the contextual signals provided by leaders. Proactive changes to one’s work inherently involve reorganizing job resources and demands, and the sustainability of this process generally depends on the availability of adequate supportive resources (Tims et al., 2012).

Employees who engage in substantial AI help-seeking can more readily obtain information, methods, and feedback, and convert technological disruption into an actionable problem-solving process (Geller and Bamberger, 2012). When leaders simultaneously exhibit high AI job crafting, this resource-acquisition process is more strongly modeled and contextualized, increasing the likelihood that employees will translate AI awareness into proactive changes in tasks, relationships, and cognition (Li et al., 2024). As AI job crafting increases, employees should be more likely to cross existing work-unit boundaries, establish external ties, and engage in boundary-spanning behavior. When employees can obtain problem-solving resources from AI and leader behavior provides cues of feasibility and legitimacy, the indirect association at a given level of AIA may become stronger. However, we must evaluate this pattern through conditional effects across combinations of AIA and the two moderators. Building on H3, we propose:

*H6*: AIHSB and LAIC jointly constitute boundary conditions for the association between AIA and BSB through AIC, such that the instantaneous indirect effect varies across combinations of AIA, AIHSB, and LAIC.

The theoretical model is presented in Figure 1.

**Figure 1:**
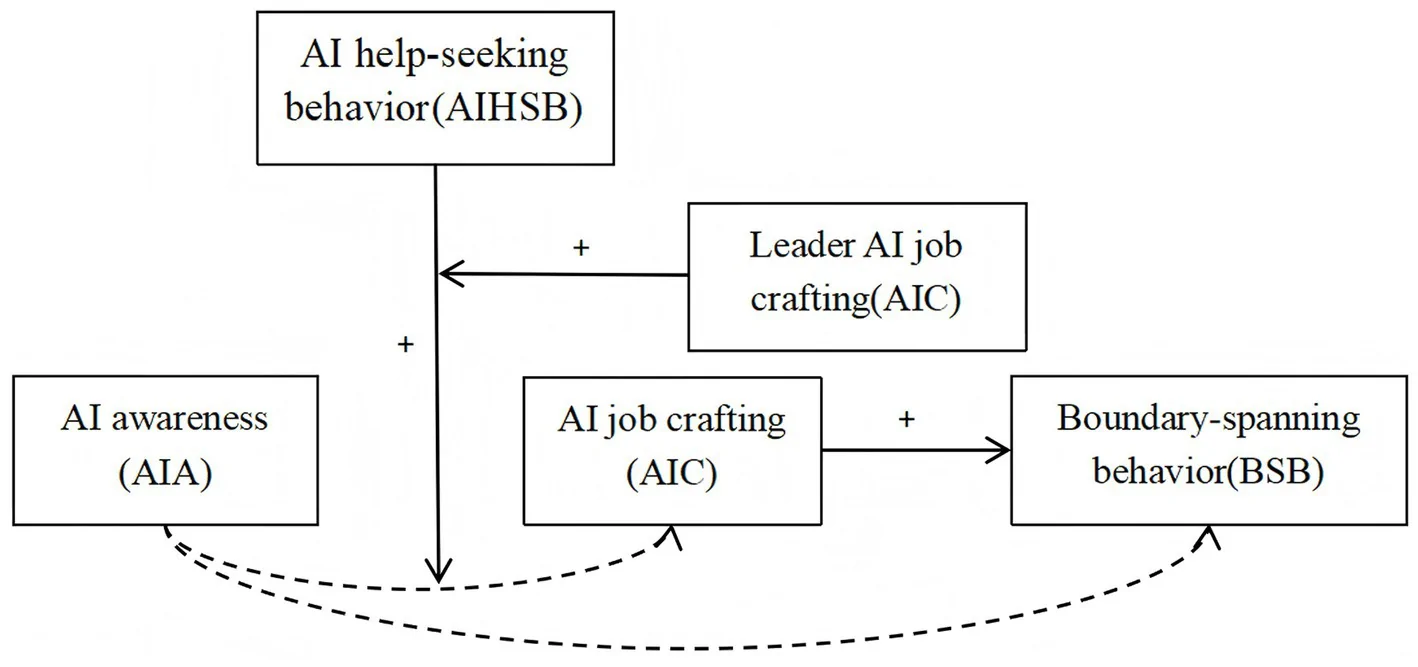
Theoretical model. Dashed arrows indicate U-shaped relationships; the solid arrow indicates a linear relationship.

## 3 Materials and methods

### 3.1 Participants

Data were collected through a survey of employees in accounting firms that had adopted AI tools. This setting was selected for two reasons. First, accounting and auditing involve substantial standardized and rule-based work, including data entry, voucher verification, information matching, risk screening, and process review. Computers and intelligent technologies are especially capable of substituting for such routine cognitive tasks, shifting labor toward nonroutine analysis, judgment, and communication (Autor et al., 2003). Second, accounting firms have embedded AI, robotic process automation, and data analytics tools into audit support, tax processing, data extraction, information retrieval, and workflow coordination (Kokina and Blanchette, 2019). These technologies streamline repetitive processes (Cooper et al., 2019) and continue to reshape the skills required in professional services (Kokina et al., 2025). The sample is therefore characterized by knowledge-intensive work, substantial task standardization, and relatively extensive use of AI, making it well-suited to testing the relationships among AI awareness, AI job crafting, and boundary-spanning behavior.

We adopted a two-wave time-lagged matched survey design. At Wave 1, participants reported their AIA, AIHSB, LAIC, and demographic information. A total of 656 valid responses were obtained at Wave 1. Approximately 2 months later, at Wave 2, participants reported their AIC and BSB. Responses across the two waves were matched using unique participant identifiers. After matching the responses across the two waves and applying data-quality screening procedures, 501 matched responses were retained for the final analyses, corresponding to a Wave 1-to-final matching rate of 76.4%. Women constituted 65.9% of the sample, and 59.2% of respondents were born in the 1990s. Unmarried employees accounted for 56.3%. The proportions with associate, bachelor’s, and master’s degrees were 3.6, 91.6, and 4.8%, respectively. Tenure of 3 years or less, three to 5 years, 6–10 years, and more than 10 years accounted for 39.5, 28.7, 24.0, and 7.8% of the sample. The final sample was thus relatively young and highly educated. With respect to AI-use frequency, 27.0% reported using AI sometimes, 31.8% occasionally, and 38.3% frequently, confirming that respondents had sufficient exposure to AI for the study.

### 3.2 Measures

All constructs were measured using established scales adapted from prior research. Unless otherwise indicated, items were rated on seven-point Likert scales ranging from 1 (“strongly disagree/never”) to 7 (“strongly agree/frequently”).

#### 3.2.1 AI awareness scale

AI awareness was measured using the four-item scale developed by Brougham and Haar (2018), adapted from Armstrong-Stassen’s (2001) measure of job insecurity. Cronbach’s alpha was 0.949.

#### 3.2.2 AI job crafting scale

AI job crafting was measured with the four-item scale developed by Li et al. (2024). References to “AI robots” in the original scale were replaced with “AI” to encompass a broader range of applications. Sample items were “When working with AI, I rearrange my tasks or workflows on my own so that I can collaborate with AI more effectively” and “When working with AI, I independently learn new knowledge and skills related to AI so that I can interact with it more effectively.” Cronbach’s alpha was 0.904.

#### 3.2.3 Boundary-spanning behavior scale

Boundary-spanning behavior was measured using the four-item scale developed by Marrone et al. (2007). The items were drawn from Ancona and Caldwell’s (1992) three-dimensional measure and capture central features of individual boundary spanning. The scale has also been validated in subsequent research (Chen and Li, 2020; Liu and Li, 2014; Zhao et al., 2021). Cronbach’s alpha was 0.904.

#### 3.2.4 AI help-seeking behavior scale

AI help-seeking behavior was assessed using six items adapted to the AI context from Geller and Bamberger’s (2012) autonomous and dependent help-seeking scales, with three items per dimension. Sample items were “To solve a problem more effectively, I ask AI for advice and new ideas and then solve the problem myself” and “I first seek help from AI to solve a problem rather than starting the task directly”. The original scale distinguishes autonomous from dependent help-seeking, and the two forms are empirically distinguishable. Because this study does not compare these help-seeking logics but instead examines employees’ overall use of AI to obtain information, methods, and problem-solving support during work, the primary analyses aggregate the six items into an overall AIHSB index. Cronbach’s alpha was 0.922.

#### 3.2.5 Leader AI job crafting scale

Leader AI job crafting was measured using a leader-referent version of the employee AI job-crafting scale (Li et al., 2024). The four items paralleled the structure of the employee measure. Cronbach’s alpha was 0.941.

The control variables were gender, year of birth, highest educational attainment, marital status, tenure with the current direct supervisor, and frequency of AI use. These variables may be associated with career stage, resource endowments, and exposure to AI; we therefore included them in the focal models to reduce potential demographic confounding. The robustness analyses yielded substantively consistent results with and without these controls.

### 3.3 Common method bias and discriminant validity

Because the study may be susceptible to common method bias, we assessed it using both Harman’s single-factor test and a common latent method factor (CLF). In Harman’s test, the first factor accounted for 24.217% of the variance, below the 40% threshold. We then conducted confirmatory factor analysis in R and added a CLF to the theoretical six-factor measurement model. The CLF model fit the data well (*χ^2^* = 131.200, *df* = 118, CFI = 0.998, RMSEA = 0.015, SRMR = 0.017). Although adding the method factor produced a statistically significant improvement in fit (*Δχ^2^* = 46.748, *Δdf* = 19, *p* < 0.001), the mean absolute standardized loading on the method factor was only 0.089, corresponding to an average explained variance of approximately 1.21%. The mean absolute change in theoretical factor loadings after adding the method factor was only 0.006, and the maximum change was 0.027. Taken together, the data contain some common-method variance, but it explains only a small proportion of variance and has limited effects on the theoretical factor loadings. Common method bias therefore does not seriously threaten the findings.

For discriminant validity, Table 1 shows that the six-factor model, in which AIA, AIC, BSB, dependent AI help-seeking (DEP), autonomous AI help-seeking (AUTO), and LAIC were modeled as distinct factors, fit the data well and significantly outperformed the five-factor, four-factor, and single-factor alternatives. The focal constructs therefore exhibit adequate discriminant validity.

**Table 1:** Comparison of confirmatory factor analysis, competing models, and the common latent method factor model.

Model	χ^2^	*χ^2^/df*	RMSEA	RMSEA 90%CI	SRMR	CFI	TLI	IFI	*Δχ^2^*	*Δdf*	*p*
Six-factor model (AIA, AIC, BSB, DEP, AUTO, LAIC)	177.95	1.299	0.024	[0.012, 0.034]	0.019	0.995	0.994	0.995	–	–	–
Five-factor model (AIA, AIC, BSB, DEP + AUTO, LAIC)	778.20	5.480	0.095	[0.088, 0.101]	0.042	0.919	0.903	0.919	600.25	5	<0.001
Four-factor model (DEP + AUTO; AIC + LAIC; AIA; BSB)	2119.58	14.518	0.164	[0.158, 0.171]	0.113	0.749	0.706	0.750	1941.63	9	<0.001
Single-factor model	6222.05	40.935	0.283	[0.277, 0.289]	0.250	0.229	0.133	0.231	6044.10	15	<0.001
CLF model	131.200	1.112	0.015	–	0.017	0.998	0.998	–	46.748	19	<0.001

“+” indicates that the corresponding constructs were combined into a single latent variable. The six-factor model is the theoretical baseline model. For the five-factor, four-factor, and single-factor models, Δ*χ^2^* and Δdf are calculated relative to the six-factor model. The CLF model adds a common latent method factor to the six-factor theoretical model; its Δ*χ^2^* and Δdf are likewise calculated relative to the six-factor model.

### 3.4 Descriptive statistics and correlations

Descriptive statistics and correlations were computed in R and are reported in Table 2.

**Table 2:** Descriptive statistics and correlations.

Variable	1	2	3	4	5	6	7	8	9	10	11	12
1. Gender	1											
2. Year of birth	−0.012	1										
3. Education	−0.014	−0.022	1									
4. Marital status	−0.002	−0.011	−0.009	1								
5. Tenure with current direct supervisor	−0.043	0.018	−0.021	−0.008	1							
6. AI use frequency	0.019	0.014	0.023	−0.001	−0.057	1						
7. AIA	−0.038	0.033	−0.026	−0.056	0.019	−0.027	1					
8. AIA^2^	−0.047	0.033	0.016	−0.082	−0.004	−0.070	0.167^***^	1				
9. AIC	−0.048	0.044	0.065	0.049	0.008	0.169^***^	−0.132^**^	0.098^*^	1			
10. BSB	0.023	−0.026	0.012	0.038	−0.031	0.072	−0.118^**^	0.159^***^	0.554^***^	1		
11. AIHSB	−0.065	−0.012	0.010	0.070	−0.002	0.135^**^	−0.023	−0.032	0.044	−0.008	1	
12. LAIC	−0.027	0.019	0.119^**^	−0.001	0.008	−0.005	0.057	0.034	0.131^**^	0.036	0.043	1
M	1.659	1994.034	2.012	1.437	2.000	3.006	3.519	0.998	5.372	5.119	4.116	5.335
SD	0.475	5.065	0.290	0.497	0.974	0.907	1.280	1.288	0.893	0.851	1.559	1.660

^*^p < 0.05, ^**^p < 0.01, ^***^p < 0.001. The same convention applies below.

Table 2 shows that AI awareness was negatively correlated with AI job crafting (*r* = −0.132, *p* < 0.01) and boundary-spanning behavior (*r* = −0.118, *p* < 0.01). The squared term of AI awareness was positively correlated with AI job crafting (*r* = 0.098, *p* < 0.05) and boundary-spanning behavior (*r* = 0.159, *p* < 0.001). AI job crafting was positively correlated with boundary-spanning behavior (*r* = 0.554, *p* < 0.001). These correlations provide preliminary evidence of potential nonlinear relationships among the focal variables and motivate the subsequent hypothesis tests.

## 4 Hypothesis testing

All data processing and statistical analyses were conducted in R. Because the model contains squared terms and multiple interaction terms, all continuous variables were standardized before constructing the squared and interaction terms, thereby reducing nonessential multicollinearity. The hierarchical regression results are reported in Table 3.

**Table 3:** Hierarchical regression results.

Variable	AI job crafting				Boundary-spanning behavior	
	M1	M2	M3	M4	M5	M6
Gender	−0.112 (0.092)	−0.101 (0.092)	−0.071 (0.092)	−0.047 (0.087)	0.034 (0.094)	0.051 (0.092)
Year of birth	0.009 (0.009)	0.008 (0.009)	0.009 (0.009)	0.006 (0.008)	−0.004 (0.009)	−0.005 (0.009)
Education	0.205 (0.151)	0.195 (0.150)	0.179 (0.149)	0.096 (0.143)	0.026 (0.154)	0.013 (0.151)
Marital status	0.087 (0.088)	0.107 (0.088)	0.100 (0.087)	0.097 (0.083)	0.064 (0.090)	0.093 (0.089)
Tenure with current direct supervisor	0.018 (0.045)	0.020 (0.045)	0.022 (0.044)	0.015 (0.043)	−0.024 (0.046)	−0.021 (0.045)
AI use frequency	0.183^***^ (0.048)	0.193^***^ (0.048)	0.193^***^ (0.048)	0.183^***^ (0.046)	0.074 (0.049)	0.088 (0.049)
AIA	−0.128^**^ (0.044)	−0.149^***^ (0.044)	−0.143^**^ (0.044)	−0.097^*^ (0.043)	−0.112^*^ (0.045)	−0.143^**^ (0.045)
AIA^2^	–	0.105^**^ (0.034)	0.119^***^ (0.034)	0.131^***^ (0.034)	–	0.150^***^ (0.035)
AIHSB	–	–	−0.109 (0.057)	−0.126^*^ (0.055)	–	–
LAIC	–	–	–	0.149^**^ (0.054)	–	–
AIA × AIHSB	–	–	−0.020 (0.042)	−0.073 (0.043)	–	–
AIA^2^ × AIHSB	–	–	0.115^***^ (0.034)	0.109^**^ (0.035)	–	–
AIA × LAIC	–	–	–	0.006 (0.045)	–	–
AIA^2^ × LAIC	–	–	–	−0.039 (0.039)	–	–
AIHSB×LAIC	–	–	–	−0.350^***^ (0.054)	–	–
AIA × AIHSB×LAIC	–	–	–	−0.012 (0.046)	–	–
AIA^2^ × AIHSB×LAIC	–	–	–	0.210^***^ (0.037)	–	–
*R^2^*	0.056	0.073	0.095	0.198	0.021	0.057
Adjusted *R^2^*	0.042	0.058	0.074	0.170	0.007	0.042
*F*	4.156^***^	4.855^***^	4.645^***^	7.012^***^	1.508	3.712^***^
Δ*R^2^*	–	0.017	0.021	0.103	–	0.036
Δ*F*	–	9.260^**^	3.860^**^	10.373^***^	–	18.758^***^

Cells report B followed by (SE). Core continuous variables were standardized using a common procedure before reconstructing the quadratic and interaction terms. ^*^p < 0.05, ^**^p < 0.01, ^***^p < 0.001.

Models M1 and M2 test the linear and nonlinear effects of AI awareness on AI job crafting. M3 tests the moderating role of AI help-seeking behavior, M4 tests the moderated moderation involving leader AI job crafting, and M5 and M6 test the linear and nonlinear effects of AI awareness on boundary-spanning behavior.

### 4.1 Main effects testing

Models M5 and M6 in Table 3 show that, after controlling for gender, year of birth, education, marital status, tenure with the supervisor, and frequency of AI use, AIA (*X*) was negatively associated with BSB (*Y*) in M5 (*B* = −0.112, *p* < 0.05). After the squared term for AIA (*X^2^*) was added in M6, the linear coefficient for AIA remained significantly negative (*B* = −0.143, *p* < 0.01). In contrast, the coefficient for the squared term was significantly positive (*B* = 0.150, *p* < 0.001). Because a significant quadratic term alone is insufficient to establish a U-shaped relationship, we followed Lind and Mehlum (2010) by testing the slopes at both ends of the observed range, the turning point, and its confidence interval. Table 4 presents the results.

**Table 4:** Formal U-shape tests and turning-point comparison for nonlinear relationships involving AI awareness.

Relationship	Linear term *b₁*	Quadratic term *b₂*	Observed AIA range	Turning point (TP)	TP 95%CI	Slope at lower bound	Slope at upper bound	U-test *p*	Conclusion
AIA → BSB (H1)	−0.143	0.150^***^	[−1.968, 2.720]	0.475	[0.147, 1.244]	−0.734 *p* = <0.001	0.675 *p* = <0.001	<0.001	Strict U-shape
AIA → AIC (H2)	−0.149	0.105^**^	[−1.968, 2.720]	0.713	[0.191, 3.488]	−0.561 *p* = <0.001	0.420 *p* = 0.012	0.012	Strict U-shape

Endpoint-slope p values are based on one-tailed tests. The 95% confidence interval for the turning point (TP) was obtained using 5,000 bootstrap resamples with the percentile method.

The standardized observed range of AIA was [−1.968, 2.720]. The turning point in the relationship between AIA and BSB was 0.475 (bootstrap 95% CI = [0.147, 1.244]). The slope at the lower bound was −0.734 (one-tailed *p* < 0.001), whereas the slope at the upper bound was 0.675 (one-tailed *p* < 0.001); the formal U-test yielded *p* < 0.001. H1 was therefore supported: the relationship was strictly U-shaped. This pattern is consistent with the resource-protection and resource-investment logic of COR theory. As Figure 2 illustrates, boundary-spanning behavior declines as AI awareness rises from low to moderate levels and begins to increase once AI awareness exceeds the turning point. At low to moderate levels of AI awareness, employees appear more likely to protect resources and contract their boundaries. At higher levels, they become more likely to move beyond established work boundaries to acquire external information, support, and resources. H1 is therefore supported.

**Figure 2:**
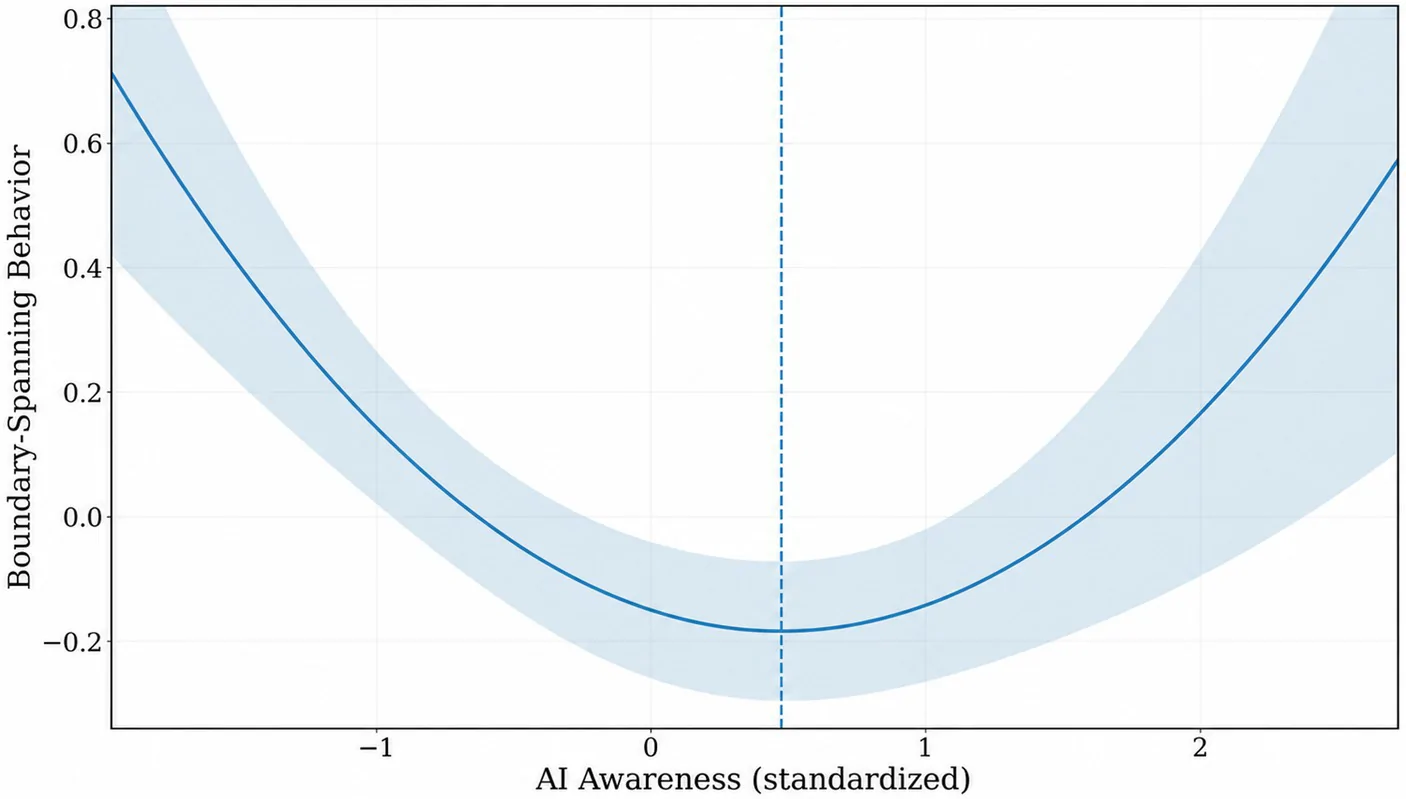
U-shaped relationship between AI awareness and boundary-spanning behavior.

Model M2 in Table 3 shows that, after the controls were included, the linear coefficient linking AIA to AIC was significantly negative (*B* = −0.149, *p* < 0.001). In contrast, the coefficient for squared AIA was significantly positive (*B* = 0.105, *p* < 0.01). As reported in Table 4, over the same observed range, the turning point in the relationship between AIA and AIC was 0.713 (bootstrap 95% CI = [0.191, 3.488]). The lower-bound slope was −0.561 (one-tailed *p* < 0.001), and the upper-bound slope was 0.420 (one-tailed *p* = 0.012); the formal U-test yielded *p* = 0.012. H2 was therefore supported: the relationship was strictly U-shaped. Figure 3 shows that AI job crafting declines as AI awareness rises from low to moderate levels, but the effect shifts from inhibition to facilitation at higher levels. Employees experiencing low to moderate AI awareness appear to prioritize resource defense and maintenance of existing practices; once awareness becomes sufficiently high, proactively revising task, relational, and cognitive boundaries becomes an important means of adapting to AI. H2 is therefore supported.

**Figure 3:**
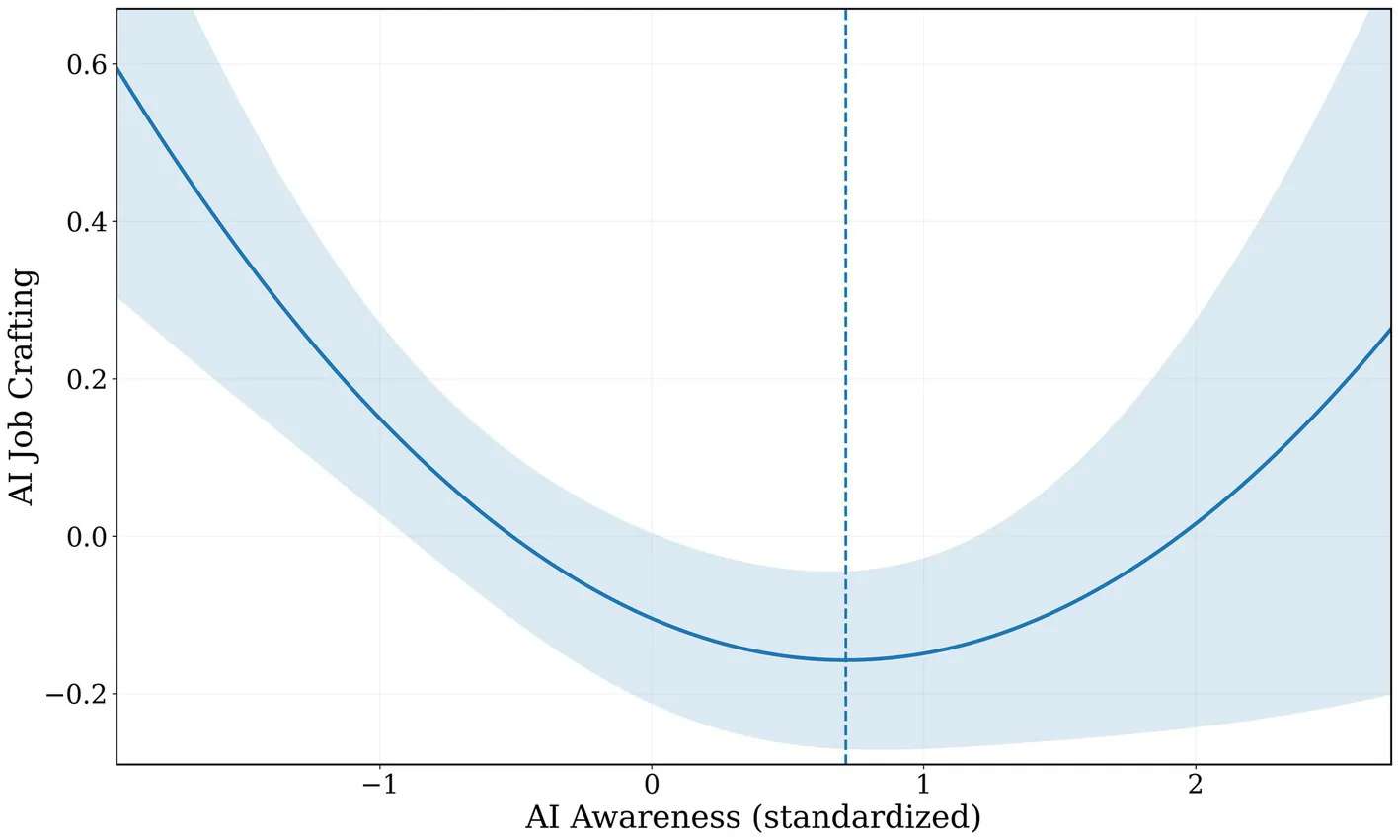
U-shaped relationship between AI awareness and AI job crafting.

Figures 2 and 3 show that although AIA had U-shaped relationships with both BSB and AIC, the estimated turning points differed. The point estimates were 0.475 for BSB and 0.713 for AIC, corresponding to different AIA levels. However, the bootstrap analysis in Table 5 estimated the difference between the two turning points as 0.238, with a 95% confidence interval of [−0.325, 2.714], which includes zero. Thus, although the sample exhibits a descriptive difference between the turning points, this difference is not statistically significant. The evidence does not support a stable difference in the overall AIA level at which the two behaviors reverse direction. Moreover, the turning points indicate AIA values associated with different outcome variables, not the time at which the behaviors occur. We therefore draw no inference about the temporal ordering of the onset of BSB and AIC.

**Table 5:** Bootstrap test of the difference between the two turning points.

Comparison	Difference *D*	95%CI	Conclusion
TP_AIC − TP_BSB	0.238	[−0.325, 2.714]	Not significant

*D* = TP_AIC − TP_BSB. Because a small number of extreme bootstrap resamples can inflate the bootstrap SE for ratio-type turning points, significance is evaluated primarily by whether the percentile 95% CI includes zero.

### 4.2 Mediation testing

Models M2 and M6 show that squared AIA was positively associated with both AIC (*B* = 0.105, *p* < 0.01) and BSB (*B* = 0.150, *p* < 0.001). Given the nonlinear relationships proposed in H1 and H2, we next examined AIC’s nonlinear mediating role. Because the relationship between AIA and AIC is quadratic, we followed Hayes and Preacher's (2010) framework for nonlinear mediation and specified separate equations for the mediator and the outcome:

$$M=\alpha_{0}+\alpha_{1}X+\alpha_{2}X^{2}+\gamma^{\prime }C+\varepsilon_{M}$$

$$Y=\beta_{0}+\beta_{1}X+\beta_{2}X^{2}+\mathrm{bM}+\delta^{\prime }C+\varepsilon_{Y}$$

Here, *X* denotes AIA, *M* denotes AIC, *Y* denotes BSB, and *C* is the vector of control variables. Because the marginal effect of AIA on AIC varies with the level of AIA, the instantaneous indirect effect through AIC at a given value x of AIA is:

$$\mathrm{IE}(x)=(\alpha_{1}+2\alpha_{2}x)b$$

We calculated IE (x) at five representative levels (−2 SD, −1 SD, the mean, +1 SD, and +2 SD) and used 5,000 bootstrap resamples to obtain 95% confidence intervals. Tables 6 and 7 reports the results. The instantaneous indirect effect through AIC varied markedly across AIA levels. At −2 SD, −1 SD, and the mean, the instantaneous indirect effects were −0.308, −0.194, and −0.081, respectively; none of their 95% bootstrap confidence intervals included zero, indicating significant negative indirect associations. At +1 SD and +2 SD, the instantaneous indirect effects became positive (0.033 and 0.146, respectively). Their 95% confidence intervals, [−0.086, 0.134] and [−0.060, 0.327], included zero, so the evidence does not establish stable positive indirect effects at high levels of AIA. The transmission effect of the quadratic term through AIC was *a*₂*b* = 0.057, with a 95% bootstrap CI of [0.010, 0.101], indicating a significant nonlinear component in how the indirect effect changes across levels of AIA.

**Table 6:** Nonlinear conditional indirect effects via AI job crafting.

AIA level	IE (*x*)	Boot SE	LLCI	ULCI	Result
−2 SD	−0.308	0.095	−0.494	−0.121	Significant
−1 SD	−0.194	0.052	−0.298	−0.095	Significant
Mean	−0.081	0.027	−0.135	−0.030	Significant
+1 SD	0.033	0.056	−0.086	0.134	Not significant
+2 SD	0.146	0.100	−0.060	0.327	Not significant

IE (*x*) = (*a₁* + *2a₂x*) × *b*; Bootstrap = 5,000.

**Table 7:** Auxiliary test of quadratic-term transmission.

Test	Effect	Boot SE	LLCI	ULCI	Result
a_2_ × b	0.057	0.023	0.010	0.101	Significant

a₂ × b is reported only as auxiliary evidence for the transmission of the quadratic term and does not substitute for the conditional indirect effect that varies across levels of AIA.

Overall, AIA has a level-dependent indirect association with BSB through AIC. The direction and significance of this association vary with AIA level; it is not stable across the full range of AIA. Employees’ perceptions of AI-related disruption are therefore associated not only directly with subsequent BSB, but also with how actively they adjust their task arrangements, collaboration patterns, and work-related cognition, which in turn is associated with whether they cross existing work-unit boundaries to seek external information and collaborative resources. AIC is thus an important transmitting variable between AIA and BSB, and its role is distinctly level dependent. H3 received conditional support.

### 4.3 Moderation testing

In Model M3 of Table 3, the interaction between squared AIA and AIHSB was significantly positive (*B* = 0.115, *p* < 0.001), indicating that AIHSB significantly moderated the positive curvature of the relationship between AIA and AIC. To identify the form of the relationship at different levels of AIHSB, we calculated the conditional effect of squared AIA at low (−1 SD), mean, and high (+1 SD) AIHSB. Table 8A reports the results.

**Table 8A:** Conditional quadratic effects at different levels of AI help-seeking behavior.

AIHSB level	Conditional quadratic term	SE	*p*	Curvature pattern
Low (−1 SD)	0.004	0.045	0.927	Nonsignificant curvature
Mean	0.119	0.034	<0.001	Significant positive curvature
High (+1 SD)	0.234	0.051	<0.001	Significant positive curvature

Table 8A shows that when AIHSB was low, the conditional quadratic term was close to zero and nonsignificant, indicating weak quadratic curvature in the relationship between AIA and AIC. At the mean and high levels of AIHSB, the conditional quadratic terms were significantly positive and increased from 0.119 to 0.234. Thus, higher AIHSB strengthened the relationship’s positive curvature.

Figure 4 further shows that when employees seldom sought information, methods, or feedback from AI, the relationship between changes in AIA and AIC was relatively flat. Even if employees increasingly recognized that AI could change their work, limited and discontinuous use of AI resources made it difficult to translate this appraisal into substantial changes in tasks, relationships, or cognition. As AIHSB increased, employees could use AI more frequently to interpret task problems, generate possible solutions, and refine their responses through feedback. The resource threat implied by rising AIA could therefore be translated more readily into specific, actionable problems. At low to moderate levels of AIA, access to these resources was not sufficient to fully offset employees’ defensive orientation. Once AIA became high, however, continued help-seeking from AI reduced the informational constraints and trial-and-error costs involved in exploring new ways of working. Employees could then respond to technological change more readily through AIC, producing a more pronounced upturn in the latter part of the curve and a clearer U-shaped relationship between AIA and AIC. H4 was supported. These results are also consistent with recent studies showing that AI knowledge and AI-related resources can shape job-crafting processes (He et al., 2024; Sha and Chai, 2025).

**Figure 4:**
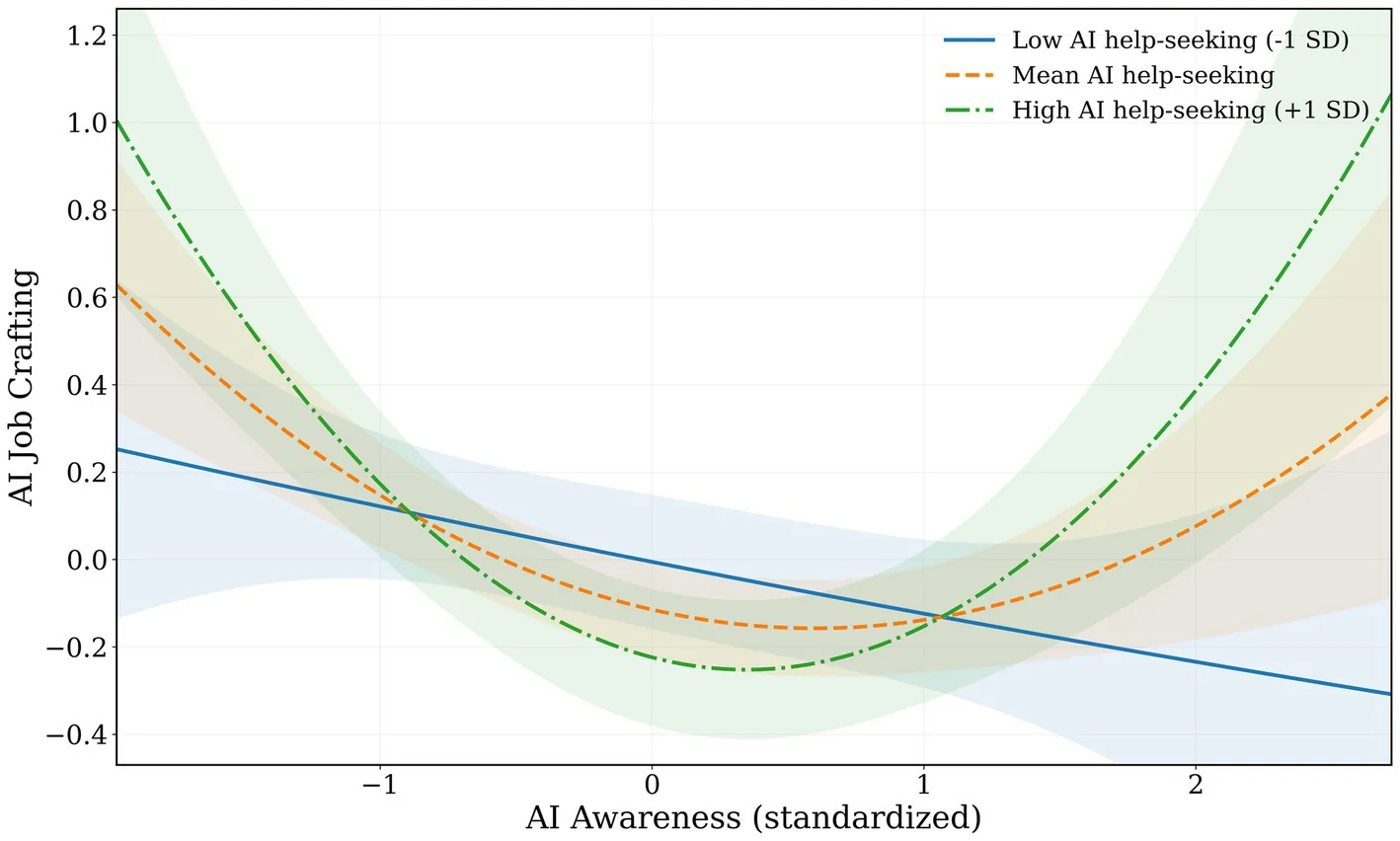
Moderating effect of AI help-seeking behavior.

In Model M4 of Table 3, after LAIC was included, the interaction between squared AIA and AIHSB remained significantly positive (*B* = 0.109, *p* < 0.01), and the three-way interaction among squared AIA, AIHSB, and LAIC was significantly positive (*B* = 0.210, *p* < 0.001). LAIC therefore significantly altered how AIHSB moderated the nonlinear curvature. To identify the form of the relationship across resource–context combinations, we set AIHSB and LAIC at low (−1 SD) and high (+1 SD) levels. We calculated the conditional effect of squared AIA. Table 8B reports the results.

**Table 8B:** Conditional curvature under different combinations of AI help-seeking behavior and leader AI job crafting.

AIHSB	LAIC	Conditional quadratic term	Curvature pattern
Low	Low	0.271^***^	Significant positive curvature
High	Low	0.069	Nonsignificant curvature
Low	High	−0.227^***^	Significant negative curvature
High	High	0.412^***^	Significant positive curvature

Table 8B shows that the joint moderation did not reduce to a simple advantage of matching levels. When both AIHSB and LAIC were low, the conditional quadratic term was significantly positive. When AIHSB was high and LAIC was low, the quadratic curvature was nonsignificant. When AIHSB was low and LAIC was high, the conditional quadratic term was significantly negative. When both variables were high, the conditional quadratic term attained its largest positive value (*B_X_^2^* = 0.412, *p* < 0.001). Taken together, these results indicate that the three-way moderation exhibits a relatively complex conditional pattern rather than a simple monotonic configuration across AIHSB–LAIC combinations.

Figure 5 also indicates that when LAIC was high, increasing AIHSB from low to high shifted the quadratic term from negative to markedly positive. In other words, the more extensively employees drew on AI resources, the more pronounced the U-shaped relationship between AIA and AIC became. By contrast, when LAIC was low, increasing AIHSB did not strengthen the curvature to the same extent. The central role of LAIC is therefore to shape whether help-seeking resources can be translated effectively into AIC, rather than merely to create a simple high–high match with employee AIHSB.

**Figure 5:**
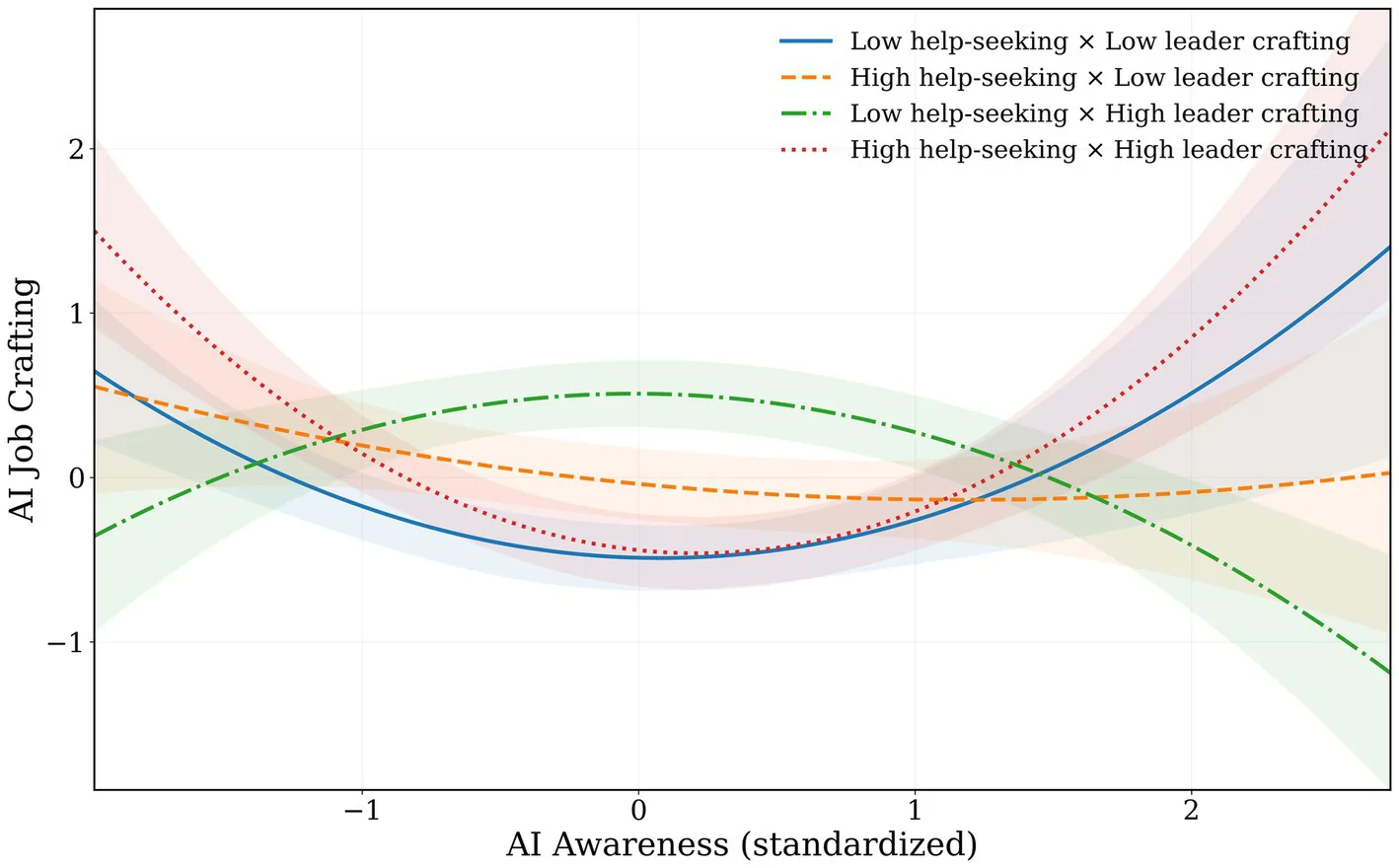
Three-way moderating effect under alternative conditions.

High LAIC may provide employees with clear cues that AI-related work adjustments are feasible and organizationally legitimate. In this context, employees who can also draw extensively on AI for information and feedback are more likely, at high levels of AIA, to translate external disruption into changes in tasks, relationships, and cognition, thereby producing the strongest positive curvature. Notably, when employee AIHSB was low and LAIC was high, the conditional curvature became negative. Leader role modeling alone may therefore be insufficient for employees to adapt to AI. If employees lack the capacity to mobilize relevant AI resources, strong leader role modeling may create a gap between demonstrated practices and employees’ own capacity to respond, preventing the expected rebound in AIC. Taken together, LAIC and employee AIHSB exhibit joint dependence between resource use and contextual role modeling, rather than a simple level-congruence effect. H5 was supported.

### 4.4 Moderated mediation testing

Building on the nonlinear mediation model, we next examined whether AIHSB and LAIC jointly altered the indirect association between AIA and BSB through AIC. Because Model M4 included the linear and squared terms of AIA and their interactions with AIHSB and LAIC, the conditional instantaneous indirect effect was expressed as follows:

$$\begin{array}{l} \mathrm{IE}(x,w,z)=b(\alpha_{1}+2\alpha_{2}x+\alpha_{5}w+2\alpha_{6}\mathrm{xw}\\ +\alpha_{7}z+2\alpha_{8}\mathrm{xz}+\alpha_{10}\mathrm{wz}+2\alpha_{11}\mathrm{xwz}) \end{array}$$

Here, w denotes AIHSB, and z denotes LAIC. Coefficients *α*₁, *α*₂, *α*₅, *α*₆, *α*₇, *α*₈, *α*₁₀, and *α*₁₁ correspond, respectively, to *X, X^2^, XW, X^2^W, XZ, X^2^Z, XWZ*, and *X^2^WZ* in Model M4; b is the coefficient linking AIC to BSB. The conditional instantaneous indirect effect therefore depends jointly on the specific levels of AIA, AIHSB, and LAIC. We set AIHSB and LAIC at −1 SD, the mean, and +1 SD, and used 5,000 bootstrap resamples to obtain 95% confidence intervals. Table 9 reports the results.

**Table 9:** Instantaneous indirect effects under representative conditions and between-group differences.

Panel A. Instantaneous indirect effects under representative conditions							
AIHSB	LAIC	AIA	IE	Boot SE	LLCI	ULCI	Result
Low	Low	Low	−0.317	0.086	−0.510	−0.171	Significant
Low	Low	High	0.271	0.124	0.025	0.511	Significant
High	Low	Low	−0.164	0.097	−0.371	0.012	Not significant
High	Low	High	−0.014	0.075	−0.152	0.145	Not significant
Low	High	Low	0.243	0.117	0.030	0.483	Significant
Low	High	High	−0.250	0.086	−0.400	−0.070	Significant
High	High	Low	−0.542	0.115	−0.771	−0.327	Significant
High	High	High	0.351	0.101	0.163	0.560	Significant

Panel B. Bootstrap tests of key between-group differences at high AI awareness				
Comparison	*ΔIE*	LLCI	ULCI	Conclusion
High–high—low–low	0.079	−0.238	0.419	Not significant
High–high—high–low	0.364	0.107	0.621	Significant difference
High–high—low–high	0.601	0.321	0.863	Significant difference

Low and high denote −1 SD and +1 SD, respectively. Panel A reports instantaneous indirect effects under representative conditions. Panel B reports differences in indirect effects across AIHSB × LAIC combinations when AI awareness is high (+1 SD). Significance is determined by whether the bootstrap 95% confidence interval includes zero.

Panel A of Table 9 reveals substantial heterogeneity in conditional instantaneous indirect effects across resource–context combinations. Under low AIHSB and low LAIC, the indirect effect shifted from significantly negative at low AIA to significantly positive at high AIA. Under high AIHSB and low LAIC, neither endpoint indirect effect was significant. Under low AIHSB and high LAIC, the indirect effect shifted from significantly positive at low AIA to significantly negative at high AIA. When both AIHSB and LAIC were high, the indirect effect shifted from significantly negative to significantly positive, exhibiting the clearest reversal in direction.

Panel B of Table 9 further compares the indirect effects across resource–context combinations when AIA was high. The indirect effect under high AIHSB and high LAIC was significantly greater than those under the two mismatched combinations—high AIHSB with low LAIC, and low AIHSB with high LAIC—but did not differ significantly from the effect under low AIHSB and low LAIC. H6 was therefore supported. AIHSB and LAIC jointly constitute boundary conditions for the association between AIA and BSB through AIC, and the conditional instantaneous indirect effects differ significantly across combinations. However, the high-AIHSB, high-LAIC combination was not significantly superior in every pairwise comparison.

### 4.5 Robustness tests

To assess whether the main conclusions depended on the control-variable specification or standard-error estimation, we estimated models without controls, models in which categorical variables were dummy coded, and models using HC3 heteroskedasticity-robust standard errors. Table 10 reports the results. Across all models, the direction and significance of AIA^2^ → AIC, AIA^2^ × AIHSB, AIA^2^ × AIHSB × LAIC, and AIA^2^ → BSB were consistent with those in the main models. The conclusions therefore did not depend on the treatment of controls or the method of estimating standard errors.

**Table 10:** Robustness checks.

Model specification	Focal effect	*B*	SE	*t*	*p*	*R^2^*	Conclusion
Main model: OLS with controls	AIA^2^ → AIC	0.105	0.034	3.043	0.002	0.073	Consistent
No controls	AIA^2^ → AIC	0.096	0.035	2.758	0.006	0.032	Consistent
Dummy-coded controls	AIA^2^ → AIC	0.105	0.035	3.031	0.003	0.079	Consistent
HC3 robust SE	AIA^2^ → AIC	0.105	0.045	2.336	0.020	0.073	Consistent
Main model: OLS with controls	AIA^2^ × AIHSB	0.115	0.034	3.384	<0.001	0.095	Consistent
No controls	AIA^2^ × AIHSB	0.116	0.034	3.387	<0.001	0.056	Consistent
Dummy-coded controls	AIA^2^ × AIHSB	0.109	0.034	3.187	0.002	0.098	Consistent
HC3 robust SE	AIA^2^ × AIHSB	0.115	0.049	2.366	0.018	0.095	Consistent
Main model: OLS with controls	AIA^2^ × AIHSB×LAIC	0.210	0.037	5.752	<0.001	0.198	Consistent
No controls	AIA^2^ × AIHSB×LAIC	0.222	0.037	6.048	<0.001	0.167	Consistent
Dummy-coded controls	AIA^2^ × AIHSB×LAIC	0.213	0.037	5.790	<0.001	0.202	Consistent
HC3 robust SE	AIA^2^ × AIHSB×LAIC	0.210	0.039	5.335	<0.001	0.198	Consistent
Main model: OLS with controls	AIA^2^ → BSB	0.150	0.035	4.331	<0.001	0.057	Consistent
No controls	AIA^2^ → BSB	0.142	0.034	4.128	<0.001	0.046	Consistent
Dummy-coded controls	AIA^2^ → BSB	0.147	0.035	4.187	<0.001	0.062	Consistent
HC3 robust SE	AIA^2^ → BSB	0.150	0.043	3.475	0.001	0.057	Consistent
DEP instead of AIHSB	AIA^2^ × DEP	0.107	0.035	3.083	0.002	0.091	Consistent
DEP instead of AIHSB	AIA^2^ × DEP × LAIC	0.206	0.038	5.424	<0.001	0.180	Consistent
AUTO instead of AIHSB	AIA^2^ × AUTO	0.103	0.033	3.076	0.002	0.091	Consistent
AUTO instead of AIHSB	AIA^2^ × AUTO×LAIC	0.172	0.035	4.844	<0.001	0.181	Consistent

The main model is estimated by OLS with demographic control variables. Dummy-coded controls indicates that categorical control variables were recoded as dummy variables. HC3 robust SE indicates re-estimation using HC3 heteroskedasticity-robust standard errors. DEP and AUTO denote dependent AI help-seeking and autonomous AI help-seeking, respectively. “Consistent” indicates that the direction and significance of the focal effect are consistent with those in the main model.

Because the overall AIHSB measure comprises items capturing both autonomous AI help-seeking (AUTO) and dependent AI help-seeking (DEP), we also re-estimated the moderation models by replacing the overall AIHSB measure with DEP and AUTO in turn. The focal two-way and three-way interaction terms remained significantly positive in both model sets. The main conclusions therefore did not depend on the specific aggregation used to construct the overall AIHSB measure.

## 5 Conclusions and discussion

### 5.1 Conclusion

Drawing on conservation of resources theory, this study documents nonlinear associations of AI awareness with employee boundary-spanning behavior and AI job crafting, identifies AI job crafting as a level-dependent transmitting mechanism, and establishes AI help-seeking behavior and leader AI job crafting as joint boundary conditions. Three conclusions emerge.

First, AI awareness exhibits a nonlinear association with employee boundary-spanning behavior. As awareness rises, boundary-spanning behavior initially declines and subsequently increases. Employees do not immediately respond to AI disruption by seeking external resources; they may first enter a period of resource contraction and behavioral conservatism. Only as AI awareness intensifies does boundary-spanning behavior become an increasingly important means of obtaining external information, relational support, and adaptive resources. The association between AI awareness and boundary-spanning behavior is thus level-dependent and transitional.

Second, AIA has a level-dependent indirect association with BSB through AIC. Employees who become aware of AI-related disruption may first reconsider their task arrangements, collaboration patterns, and work-related cognition, rather than simply increasing or decreasing external collaboration and resource seeking. At low to moderate levels of AIA, employees are more likely to reduce work adjustments to protect existing resources, which may also weaken their willingness to seek external information, support, and collaborative opportunities. As AIA intensifies, employees become more likely to reconfigure the task, relational, and cognitive boundaries of their work and gradually extend these internal adjustments to broader external resource connections. AIC is therefore an important behavioral link through which employees move from awareness of technological disruption toward boundary-spanning adaptation.

Third, AIHSB and LAIC are important boundary conditions for the preceding mechanism. As AIHSB increased, the positive curvature of the relationship between AIA and AIC generally strengthened, although the magnitude of this effect also depended on LAIC. When both employee AIHSB and LAIC were high, the indirect association between AIA and BSB through AIC showed the clearest transition from negative to positive as AIA increased; however, this high–high combination did not yield the largest positive effect at every level of AIA. Overall, AIHSB provides instrumental resources, whereas LAIC provides role-modeling and legitimacy cues. Together, they shape how employees allocate resources and adapt to AI-related disruption.

### 5.2 Theoretical contributions

First, the study reconciles competing claims about whether AI awareness facilitates or inhibits employee behavior by adopting a nonlinear perspective. Prior research on challenge–hindrance appraisals has shown that employees’ appraisals of AI may be associated with different outcomes through job crafting, job insecurity, and AI knowledge (He et al., 2024; Sha and Chai, 2025). Yet, empirical explanations have generally remained linear: some emphasize insecurity, exhaustion, and withdrawal (Lingmont and Alexiou, 2020), whereas others emphasize proactive learning, work engagement, and job crafting (Ding, 2021; Kang et al., 2023). This study finds U-shaped relationships of AIA with both BSB and AIC. AIA therefore does not have uniformly positive or negative implications; different levels may correspond to distinct patterns of resource appraisal. At low to moderate levels, employees focus on resource loss and the costs of action, reducing boundary spanning and AI job crafting. At higher levels, they recognize that preserving existing task boundaries and collaborative practices may itself lead to greater resource loss, prompting proactive adjustments and external resource searches. This finding advances the double-edged-sword account of AI awareness from a linear opposition to a nonlinear transition. It enriches conservation of resources theory by showing a nonlinear pattern that is consistent with a shift from resource defense toward resource investment as AIA increases (Hobfoll et al., 2018).

Second, we extend the nonlinear theorization of AIA to mediation analysis and show that AIC’s transmitting role is also level dependent. Conventional mediation studies often treat a predictor’s indirect effect on an outcome as a constant, implicitly assuming that the relationships composing the mediating pathway are approximately linear. When AIA and AIC are U-shaped, however, a one-unit increase in AIA does not produce a constant change in AIC; the indirect association between AIA and BSB through AIC should therefore not be summarized by a single coefficient. Following Hayes and Preacher's (2010) approach to nonlinear mediation, we examined instantaneous indirect effects at different levels of AIA. We found that both their magnitude and direction varied across the range of AIA. AIC is thus not a stable transmission mechanism operating identically at every level of technological disruption. As AIA moves from lower to higher levels, the relationship shifts from a range consistent with resource defense toward one consistent with resource investment, and the direction of the indirect association changes accordingly. By moving the question of when AIA shifts from inhibition to facilitation from the outcome level to the process level, this study aligns the statistical test of mediation with the nonlinear theoretical hypothesis. It extends understanding of the dynamic and conditional mechanisms associated with AIA.

Third, the study develops and tests a dual-boundary mechanism combining instrumental resources and contextual modeling. Whether AI awareness becomes AI job crafting and, subsequently, boundary-spanning behavior depends not only on the perceived intensity of AI disruption but also on the resources employees can mobilize and on whether the organizational context legitimizes their use. AI help-seeking behavior represents an instrumental strategy for obtaining information, methods, and feedback under technological uncertainty. Leader AI job crafting provides behavioral modeling and contextual cues concerning whether AI-related adaptation is feasible, appropriate, and recognized. Related evidence shows that leader AI symbolization can stimulate employee improvisation through the positive pathway of creative self-efficacy and the negative pathway of job insecurity (Liu et al., 2026b). The present findings further show that LAIC does not simply amplify the beneficial role of AIHSB. Rather, it shapes whether and how AI help-seeking resources are translated into AI job crafting, with the form and strength of the nonlinear association varying across different AIHSB–LAIC combinations. By integrating individual access to AI-enabled resources with leader-level contextual modeling, the study shows that employee resources and organizational signals jointly condition proactive adaptation. This person–context mechanism extends research on the boundary conditions of AI job crafting and provides a more complete explanation of when employee boundary-spanning behavior is likely to emerge (Bandura, 1977; Geller and Bamberger, 2012).

### 5.3 Managerial implications

Organizations should establish dynamic mechanisms to assess employees’ AI awareness and actively guide them to navigate AI-driven career change. As AI becomes more deeply embedded in organizational processes, firms should neither minimize nor avoid its disruptive implications. They should communicate changes in jobs, demonstrate concrete AI applications, and update capability requirements so that employees understand how AI may reshape task boundaries, skill structures, and career development. Organizations should pair efforts to help employees understand AI-driven change with access to AI learning resources, help-seeking channels, and leader role modeling. Doing so may prevent employees from remaining in a resource-defense state as AIA rises, help them shift sooner from passive defense to proactive crafting and boundary spanning, and support their career adaptation in the AI era.

Managers should also cultivate an organizational context that supports AI adaptation by strengthening leader modeling and employees’ capacity to seek help from AI. Leaders should not merely instruct employees to use AI; they should consistently signal constructive engagement with AI and demonstrate how it can optimize task arrangements, improve collaboration, and increase problem-solving efficiency. When new AI tools or use cases are introduced, organizations should provide timely training on the tools, prompt-engineering practice, case-based exercises, and experience sharing. These interventions can improve employees’ ability to learn and use AI effectively and normalize AI help seeking. The combination of leader modeling and employee AI capability building is essential for promoting AI job crafting and boundary-spanning behavior.

### 5.4 Limitations and future research

Several limitations should be acknowledged. First, the sample was drawn from employees of accounting firms that had introduced AI tools. This group is characterized by knowledge-intensive work, strongly rule-based tasks, and relatively salient perceptions of technological substitution, making it appropriate for the research setting. Nevertheless, the depth of AI adoption, the form of task substitution, and the demand for boundary spanning vary across industries. The generalizability of the findings to manufacturing, internet services, education, healthcare, and the public sector therefore requires further examination. Future research should use broader cross-industry and cross-occupational samples to strengthen external validity. Second, the examination of AI awareness is based on a relatively static research design. Although the study employed a two-wave time-lagged matched survey design, AI job crafting and boundary-spanning behavior were both measured at Wave 2. Therefore, the design does not establish temporal precedence between these two variables and does not support strict causal inference regarding the mediating process. As AI technologies evolve, employees’ perceptions are likely to change with the depth of implementation, forms of organizational support, and accumulated personal experience. Multiwave panel studies, experience-sampling designs, and longitudinal models could examine how AI awareness develops over time and how these changes shape job crafting, boundary spanning, and career adaptation. Third, drawing on COR theory, this study explains the U-shaped relationships between AIA and employee behavior as a transition from resource protection to resource investment. However, we did not directly measure employees’ perceived resource loss, resource-defense orientation, willingness to invest resources, or changes in these constructs over time. Future research should incorporate perceived resource loss, resource-conservation motives, and willingness to invest resources to test more directly when and why employees shift from resource protection to resource investment.

## Data Availability

The raw data supporting the conclusions of this article will be made available by the authors, without undue reservation.
